# Patient preferences for treatment‑free intervals in multiple myeloma: mixed‑methods insights on monitoring trade‑offs and supportive care improvements

**DOI:** 10.1007/s00520-026-11255-z

**Published:** 2026-10-02

**Authors:** Anna Fleischer, Mohammad Barakat, Jessica Peter, Sofie-Katrin Kadel, Christine Riedhammer, Patrick-Pascal Strunz, Julia Mersi, Johannes Waldschmidt, K. Martin Kortüm, Hermann Einsele, Imad Maatouk, Leo Rasche

**Affiliations:** 1 Department of Internal Medicine II, University Hospital Würzburg, Würzburg, Germany; 2 Department of Internal Medicine II, Psychosomatics, University Hospital Würzburg, Oberdürrbacherstr str. 6, 97080 Würzburg, Germany; 3 Department of Medicine II, Rheumatology and Clinical Immunology, University Hospital Würzburg, Würzburg, Germany, Würzburg, Germany

**Keywords:** Multiple myeloma, Treatment-free intervals, Patient preferences, Monitoring, Supportive care, Outpatient follow-up

## Abstract

**Purpose:**

Continuous and maintenance therapy remains central to multiple myeloma (MM) care, but repeated treatment-related healthcare encounters may impose physical, psychosocial, and logistical burden. We explored patient preferences regarding intervals between active treatment contacts, monitoring trade-offs, and opportunities to improve inpatient and outpatient care.

**Methods:**

A 12-month, single-center mixed-methods survey was conducted among 100 patients with MM at a German tertiary center. The survey assessed the perceived importance and preferred length of treatment-free intervals (TFIs), monitoring perceptions, relapse-risk trade-offs, inpatient stressors, and care-improvement proposals. Quantitative responses were summarized with item-specific denominators and exact 95% confidence intervals (CIs). Free-text responses underwent inductive content analysis by two independent raters and mixed-methods integration.

**Results:**

Intervals between inpatient treatments were rated 10/10 by 49/100 participants (49.0%; 95% CI 38.9–59.2) and 0/10 by 9/100 (9.0%; 95% CI 4.2–16.4). The most frequently reported inpatient stressors were separation from family (27.0%), treatment-related symptoms or side effects (23.0%), and fatigue (17.0%). Regular check-ups were reassuring for 77/100 (77.0%; 95% CI 67.5–84.8) and burdensome for 13/100 (13.0%; 95% CI 7.1–21.2). Seven of 100 participants (7.0%; 95% CI 2.9–13.9) explicitly accepted higher relapse risk for longer TFIs. Preferred minimum interval length was heterogeneous; 39/99 (39.4%; 95% CI 29.7–49.7) preferred > 8 weeks. Qualitative narratives emphasized recovery, autonomy, restoration of routines, and modifiable care-process factors.

**Conclusion:**

Patients frequently valued intervals between active treatment contacts for recovery, while monitoring was predominantly reassuring. These descriptive findings do not establish the safety or clinical appropriateness of longer TFIs but support prospective evaluation of preference-sensitive scheduling and monitoring.

**Supplementary information:**

The online version contains supplementary material available at https://doi.org/10.1007/s00520-026-11255-z.

## Introduction

Continuous and maintenance therapy have substantially improved outcomes in multiple myeloma (MM) and remain central to disease control [1, 2]. After autologous stem-cell transplantation, lenalidomide maintenance has improved progression-free survival [3, 4] and, in an individual-patient-data meta-analysis, overall survival [3], and contemporary transplant guidance continues to include maintenance as a standard component of post-transplant care [5]. Prior patient-centered studies in MM show that quality of life and social support are important dimensions of the treatment experience [6, 7]. At the same time, prolonged therapy, repeated healthcare encounters, and treatment-specific toxicities can impose physical, psychosocial, nutritional, and logistical burden [8–11]. In practical terms, the present survey focused on patient-experience domains that may contribute to this burden: treatment-related symptoms and fatigue; separation from family during admission; food, room, environmental, and activity conditions; waiting and communication; disruption of home, work, and social routines; travel and appointment logistics; and the potentially competing reassurance and burden of monitoring. TFIs should therefore not be understood as a general alternative to evidence-based maintenance therapy. The relevant supportive-care question is how patients perceive intervals between active treatment contacts, how they weigh such intervals against monitoring and relapse-related concerns, and which modifiable care-process factors shape treatment-related burden.

The therapeutic landscape of MM is increasingly heterogeneous, ranging from continuous or prolonged maintenance strategies to contemporary immunotherapies including bispecific antibodies and CAR T-cell therapies [1, 12, 13]. In addition to efficacy, treatment selection may involve safety, route of administration, organizational burden, and patient-relevant priorities [14]. Selected cellular therapy strategies may create prolonged periods without scheduled anti-myeloma drug administration in responding patients [15–17], although this does not remove the need for structured follow-up, supportive care, and surveillance for acute or delayed toxicities [18–21]. Likewise, infection prevention, immunoglobulin replacement, and other supportive-care contacts may remain necessary with T-cell-redirecting therapies [20, 22–24]. This distinction is important when interpreting patient preferences for treatment-free intervals.

Several patient-centered questions remain insufficiently characterized: how patients with MM value intervals between active treatment contacts; how they balance such intervals against reassurance from regular follow-up and concerns about relapse risk; and which inpatient and outpatient care factors most strongly affect perceived burden. Descriptive evidence on these preferences may help inform the design of future supportive-care and monitoring models, but cannot by itself establish the clinical safety or appropriateness of longer treatment-free intervals.

This mixed-methods survey addresses these gaps by combining structured preference items and qualitative narratives to describe the perceived importance of treatment-free intervals, characterize monitoring and relapse-risk trade-offs, and identify patient-generated suggestions for improving inpatient and outpatient care experience.

## Methods

### Design

This was a 12-month, single-center, descriptive mixed-methods survey of patients with multiple myeloma (MM) at a German tertiary center.

### Setting and participants

Patients were approached during scheduled inpatient admissions and outpatient visits at University Hospital Würzburg. Because patients were included irrespective of treatment line or disease phase, the enrolled sample comprised a heterogeneous tertiary-center MM population. Eligibility criteria were age ≥ 18 years, confirmed MM according to IMWG criteria [25], capacity to consent, and sufficient German proficiency to complete the survey. Exclusion criteria were acute psychiatric crisis, decision-relevant cognitive impairment, intensive-care admission, or insufficient language proficiency. The prespecified pragmatic recruitment target was 100 evaluable questionnaires. The number approached and reasons for nonparticipation could not be reconstructed from the retained study documentation; consequently, a participation proportion could not be calculated. Questionnaire setting (inpatient admission or outpatient visit) was recorded descriptively. A setting-stratified outcome analysis was not conducted because the exploratory study was not designed for adjusted subgroup inference and detailed treatment- and disease-related covariates were unavailable.

### Endpoints

The primary descriptive outcome was the perceived importance of treatment-free intervals.

Secondary descriptive outcomes included:

- Inpatient stressors and care experience,
- Reassurance versus burden associated with outpatient visits,
- Preferences regarding monitoring intensity and visit cadence,
- Willingness to trade interval length against relapse-risk concerns, and
- Patient-generated proposals for improving inpatient and outpatient care experience.

### Survey content

The survey instrument was specifically developed for this study, drawing on prior work on patient-reported outcomes, psychosocial factors, and treatment-specific supportive care in multiple myeloma and on clinical and psychosocial expertise within the study team [6, 7, 9, 10]. Before answering TFI-related items, participants received a brief standardized explanation. A TFI was described as a period between active anti-myeloma treatment contacts or treatment cycles during which no scheduled anti-myeloma therapy was administered. Participants were informed that such intervals could still include monitoring visits, laboratory assessments, supportive-care contacts, prophylactic interventions, or urgent reassessment if clinically indicated. Separate items addressed inpatient and outpatient contexts to avoid conflating recovery after hospitalization with intervals between ambulatory treatment appointments. Participants were asked to report the number of “inpatient days in the preceding year.” The available item wording does not establish whether all respondents consistently excluded outpatient clinic or day-case encounters; this ambiguity is therefore acknowledged as a potential source of classification error. Administration route and regimen-level details were not captured sufficiently to distinguish self-administered oral treatment from clinic-administered infusions or injections.

The instrument comprised:

- Sociodemographic variables and selected treatment-exposure variables, including self-reported inpatient days in the prior year and questionnaire setting,
- Structured items on TFI importance (0–10), preferred minimum interval length, monitoring reassurance or burden, visit cadence, and relapse-risk trade-offs,
- Structured checklists of inpatient stressors and outpatient barriers, and
- Free-text fields for patient narratives on challenges, coping strategies, care needs, and improvement proposals.

### Data collection and quality assurance

Surveys were completed on paper or via a secure hospital platform. Trained staff provided assistance when needed. Completeness was checked at collection; data were de-identified, consistency-checked, and stored on protected institutional systems with role-based access.

### Statistical analysis

Analyses were descriptive and exploratory. Continuous variables are reported as median and interquartile range (IQR), and categorical variables as n/N and percentages using item-specific denominators. For survey-response proportions and coded response categories, two-sided exact 95% confidence intervals (CIs) were calculated using the Clopper-Pearson method. Demographic and clinical characteristics were summarized descriptively without CIs. No formal hypothesis testing was performed. Missing data were handled using available-case analysis. As the available aggregate values for the visit-frequency item and the non-affirmative categories of the relapse-risk trade-off item did not reconcile with the cohort denominator, these distributions were not imputed or reported; only the directly verifiable affirmative relapse-risk response (7/100) was retained.

### Qualitative analysis

Free-text responses underwent inductive qualitative content analysis. Two independent raters coded the narratives, iteratively refined a consensus codebook, and resolved discrepancies through discussion. Codes were grouped into higher-order themes reflecting perceived burdens, coping strategies, monitoring preferences, and patient-generated improvement proposals.

### Mixed-methods integration

Mixed-methods integration was performed after separate descriptive quantitative analysis and qualitative coding. Quantitative findings from structured survey items were systematically compared with qualitative themes derived from free-text responses. Relationships between data strands were categorized as convergence, when quantitative and qualitative findings pointed in the same direction; complementarity, when qualitative narratives helped explain or contextualize quantitative patterns; and divergence, when qualitative accounts introduced nuance or tension not captured by the structured items.

### Ethics and data protection

Ethics approval was obtained from the University of Würzburg (AZ 246/22). All participants provided written informed consent. Data were de-identified and processed according to institutional policies and applicable regulations.

## Results

### Participants and baseline characteristics

A total of 100 patients with MM provided evaluable questionnaires. Median age was 67.2 years (IQR 60.8–72.3), and 64% were male. Questionnaires were completed during an inpatient admission by 45 participants and during an outpatient visit by 55. Because the number approached and reasons for nonparticipation could not be reconstructed from the retained study documentation, a participation proportion could not be reported.

Median self-reported inpatient days in the prior year were 31 (IQR 12–57). The survey item referred to “inpatient days in the preceding year”; however, its wording did not establish whether all respondents consistently distinguished inpatient days from outpatient or day-case encounters. Responses were not validated against administrative records, and planned and unplanned admissions were not distinguished; recall or classification error therefore cannot be excluded. Supplementary Table 1 summarizes demographics and available clinical exposure variables.

### Importance of TFIs

**Fig. 1:**
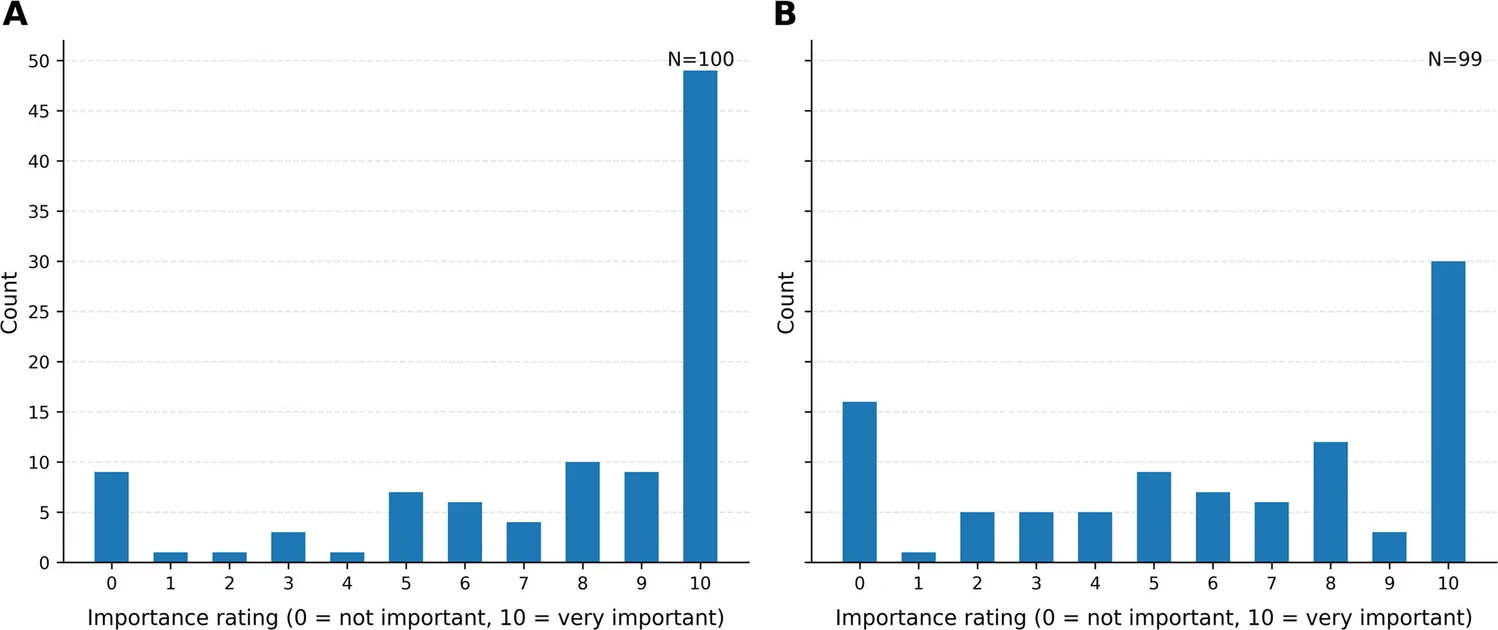
Perceived importance of treatment-free intervals in inpatient (**A**; *N* = 100) and outpatient (**B**;* N* = 99) contexts. Bars show respondent counts across 0–10 ratings. Item-specific percentages and exact 95% CIs are provided in Supplementary Table 2

In the structured survey items, intervals between inpatient treatments were rated 10/10 (“very important”) by 49/100 (49.0%; 95% CI 38.9–59.2) and 0/10 (“not important”) by 9/100 (9.0%; 95% CI 4.2–16.4). Figure 1 illustrates the count distributions, and Supplementary Table 2 provides item-level proportions with exact 95% CIs. In qualitative free-text responses, high TFI importance was commonly linked to physical and emotional recovery, family reconnection, restoration of routines, social activities, travel, and autonomy.

### Inpatient stressors and care experience

In the structured stressor items, the most frequently reported inpatient stressors were separation from family [27/100 (27.0%; 95% CI 18.6–36.8)], treatment-related symptoms or side effects [23/100 (23.0%; 95% CI 15.2–32.5)], fatigue [17/100 (17.0%; 95% CI 10.2–25.8)], food quality [15/100 (15.0%; 95% CI 8.6–23.5)], waiting times [14/100 (14.0%; 95% CI 7.9–22.4)], and shared rooms [12/100 (12.0%; 95% CI 6.4–20.0)]. Additional categories were communication or empathy gaps [11/100 (11.0%; 95% CI 5.6–18.8)], climate control [10/100 (10.0%; 95% CI 4.9–17.6)], other health-related limitations [9/100 (9.0%; 95% CI 4.2–16.4)], and lack of physical activity [7/100 (7.0%; 95% CI 2.9–13.9)]. The 9/100 category represented additional limitations not already coded as symptoms/side effects or fatigue and should not be interpreted as the prevalence of health- or disease-related distress. Stress attributable to MM itself was not assessed as a standalone category. Supplementary Table 3 provides the full categories. Effective coping strategies reported in free-text responses are summarized in Supplementary Table 4.

### Impact on daily life domains

These findings describe the perceived impact of treatment-related healthcare encounters, including inpatient admissions, outpatient appointments, and monitoring visits, on daily life; they should not be interpreted as effects of TFIs alone. Regarding home management, 67/100 (67.0%; 95% CI 56.9–76.1) reported no major backlog of home tasks, often because of planning, delegation, or online management, whereas 33/100 (33.0%; 95% CI 23.9–43.1) reported difficulties, especially with frequent admissions, cognitive strain, or stress. Social relationships were negatively affected in 32/100 (32.0%; 95% CI 23.0–42.1), mainly through cancellations, infection precautions, or exhaustion; others described maintaining or strengthening contacts through proactive planning and digital communication. Among the 42 participants not categorized as retired or early retired, 23/42 (54.8%; 95% CI 38.7–70.2) reported work-related or role-related limitations. Flexible scheduling and supportive workplace policies were described as mitigating factors.

### Outpatient TFIs, monitoring reassurance, and risk trade‑offs

**Fig. 2:**
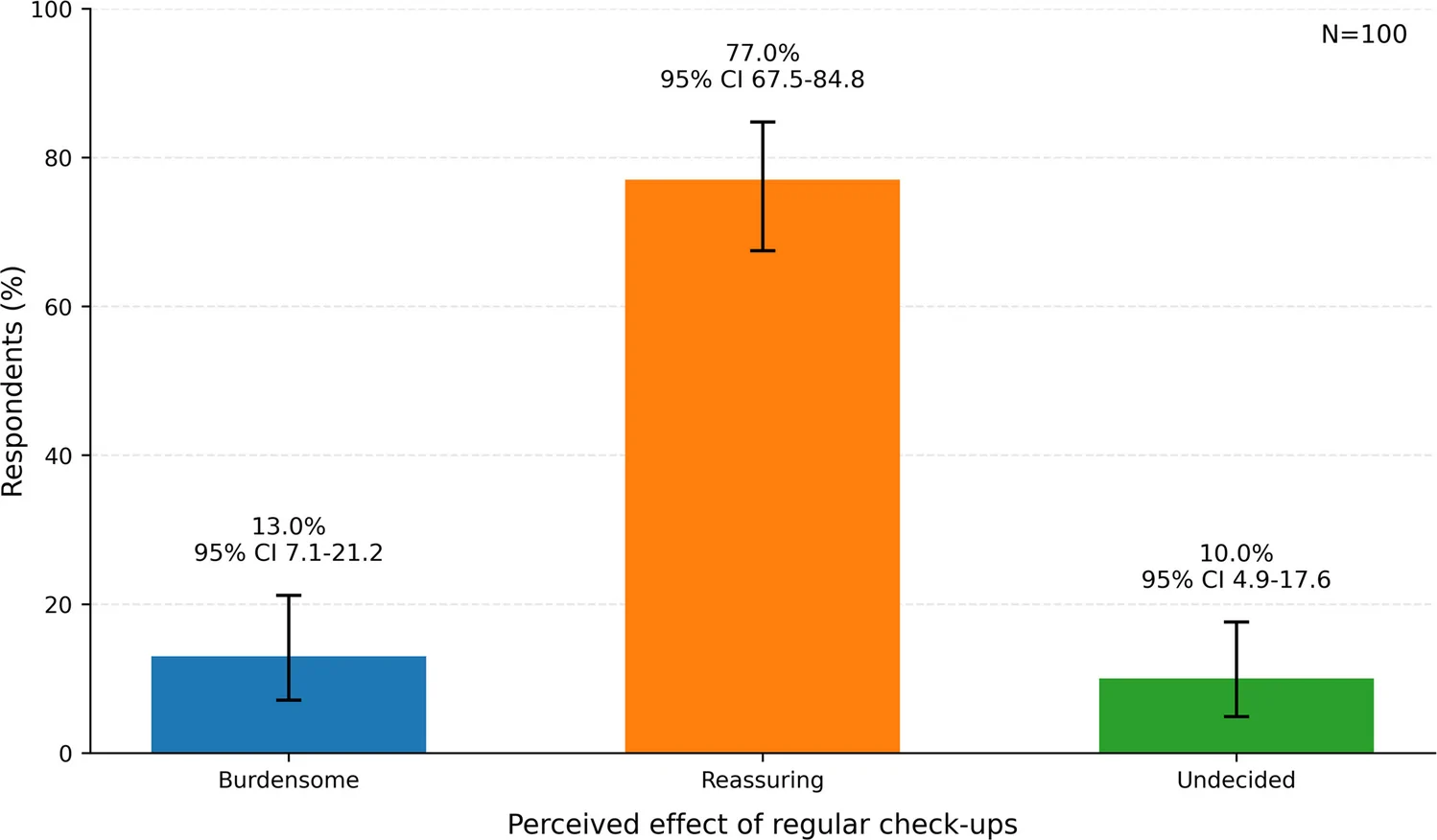
Perceived effect of regular check-ups. Bars show response percentages and error bars show two-sided exact 95% CIs (Clopper-Pearson); *N* = 100

Preferences regarding outpatient intervals between treatment-related contacts were heterogeneous: a rating of 10/10 was selected by 30/99 (30.3%; 95% CI 21.5–40.4), whereas 0/10 was selected by 16/99 (16.2%; 95% CI 9.5–24.9) (Fig. 1 and Supplementary Table 2). Regular check-ups were described as reassuring by 77/100 (77.0%; 95% CI 67.5–84.8), burdensome by 13/100 (13.0%; 95% CI 7.1–21.2), and neither/undecided by 10/100 (10.0%; 95% CI 4.9–17.6) (Fig. 2). In the relapse-risk trade-off item, 7/100 (7.0%; 95% CI 2.9–13.9) explicitly accepted a higher relapse risk in exchange for longer TFIs. The remaining response categories are not reported separately because their archived summary values did not reconcile to the cohort denominator; no corrected distribution was inferred.

### Preferred minimum TFI length

**Fig. 3:**
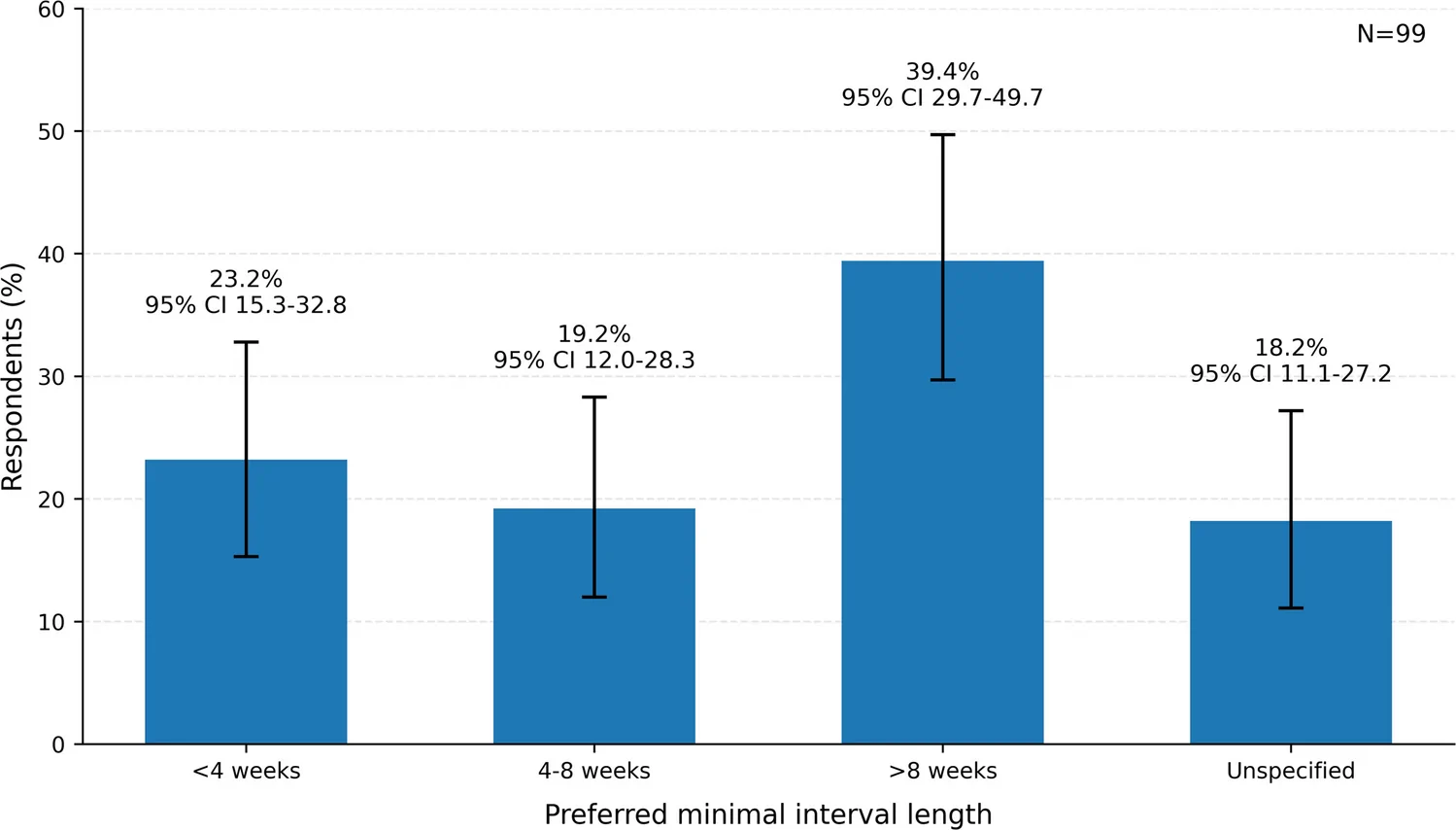
Patient-reported preferred minimum interval length between treatment-related contacts. Bars show response percentages and error bars show two-sided exact 95% CIs (Clopper-Pearson); *N* = 99

Among 99 respondents, preferred minimum intervals were < 4 weeks for 23/99 (23.2%; 95% CI 15.3–32.8), 4–8 weeks for 19/99 (19.2%; 95% CI 12.0–28.3), > 8 weeks for 39/99 (39.4%; 95% CI 29.7–49.7), and unspecified for 18/99 (18.2%; 95% CI 11.1–27.2) (Fig. 3). Qualitative explanations referenced recent treatment intensity, recovery needs, travel logistics, family and work responsibilities, anxiety about relapse, and personal circumstances.

### Mixed-methods integration

Mixed-methods integration showed convergence between high inpatient TFI importance ratings and qualitative descriptions of physical recovery, emotional relief, family reconnection, and restoration of routines. Complementarity was observed for outpatient monitoring: the predominance of reassuring responses was contextualized by narratives emphasizing predictable scheduling, clear communication, and rapid access in case of symptoms. Divergence was evident because some participants valued longer intervals between treatment-related visits, whereas others preferred frequent contact to reduce relapse-related anxiety. These patterns are descriptive and were not examined by recruitment setting or treatment route.

### Patient‑generated improvement proposals

Patient-generated improvement proposals primarily addressed inpatient and outpatient care experience rather than TFIs directly. Frequently mentioned suggestions included greater availability of single rooms and quieter room conditions, improved food quality, reduced waiting times, flexible appointment scheduling, adequate staffing, clearer communication, and access to rehabilitative activities such as physiotherapy, yoga, or low-intensity exercise. Factors affecting attendance for maintenance-therapy appointments are summarized in Supplementary Table 5.

## Discussion

This single-center mixed-methods survey describes how participants with MM perceived intervals between active treatment contacts, monitoring, and care-related burden. Intervals between inpatient treatment contacts were frequently linked to physical recovery, emotional relief, family reconnection, and restoration of daily routines. Regular monitoring was more often described as reassuring than burdensome, while only a small minority explicitly accepted higher relapse risk for longer intervals. The findings suggest that valuing intervals between active treatment contacts and valuing continued follow-up were not mutually exclusive in this sample. Predictable monitoring and access to reassessment may contribute to whether such intervals are experienced as acceptable or reassuring; however, the present study did not evaluate clinical safety.

The interpretation of these findings requires careful clinical contextualization. The enrolled cohort was slightly younger than the German real-world BasecaMMp cohort, which reported a median age of 72.5 years [26]. Continuous and maintenance therapy remain central to MM care, and lenalidomide maintenance after autologous transplantation has demonstrated a progression-free survival benefit [3, 4], with an overall survival benefit shown in an individual-patient-data meta-analysis [3]. The toxicity burden associated with corticosteroids depends on dose, schedule, treatment duration, cumulative exposure, age, frailty, and comorbidity; these regimen-level factors were not captured in the present survey [27]. Similarly, the experience of a self-administered oral regimen may differ from that of infusions or injections requiring clinic attendance. Because administration route and current regimen were not systematically recorded, the present findings cannot be attributed to a specific treatment modality and should not be interpreted as support for interrupting evidence-based therapy.

Preferences for intervals between active treatment contacts were heterogeneous. This heterogeneity is clinically plausible because preferences are likely shaped by disease phase, treatment line, current response status, prior toxicity, functional status, travel burden, family and work responsibilities, anxiety about relapse, and individual goals. More broadly, prior patient-centered MM studies have highlighted the relevance of quality of life, social support, and treatment-specific toxicities to patients’ treatment experience and supportive-care needs [6–11]. Accordingly, preferred TFI length in this study should be interpreted as a descriptive preference signal rather than as a clinically actionable recommendation.

The median of 31 self-reported inpatient days in the preceding year and the prominence of separation from family should be interpreted in the context of a German tertiary-care setting. Some treatment, monitoring, and supportive-care processes that involve admission in one health system may be delivered partly or predominantly as outpatient care in another [28–31]. In addition, 45 participants completed the questionnaire during an admission and 55 during an outpatient visit. The immediate setting may have influenced salience, recall, or response framing. A setting-stratified outcome analysis was not conducted; the pooled estimates may therefore partly reflect recruitment context and should be interpreted accordingly.

Finite-course therapies such as CAR T-cell therapy may create prolonged periods without scheduled anti-myeloma drug administration in selected responders [15–17]. However, finite-course treatment should not be equated with low-burden or low-risk care. CAR T-cell therapy can be associated with acute and delayed toxicities, including CRS, ICANS, cytopenias, infections, hypogammaglobulinemia, delayed neurological syndromes, secondary malignancies, and rare fatal complications [18, 20, 21]. Moreover, patients may require ongoing surveillance, prophylaxis, immunoglobulin replacement, and supportive-care visits [18–20]. European CAR T-cell patient-reported outcome data further underscore that mental and social well-being, working life, treatment experiences, and unmet care needs remain relevant after therapy [19]. Thus, patient-perceived TFIs after cellular therapy may be shaped not only by the absence of active anti-myeloma treatment but also by the frequency and burden of post-treatment healthcare encounters.

For bispecific antibodies, healthcare-setting requirements are evolving. Selected centers increasingly deliver step-up dosing in outpatient pathways [30, 31], and observational data suggest that prophylactic IL-6 receptor blockade with tocilizumab can reduce, although not eliminate, cytokine release syndrome during teclistamab initiation [32]. These developments may reduce hospitalization for selected patients and make inpatient burden estimates time- and system-dependent. They nevertheless require patient selection, caregiver and access planning, monitoring, infection-prevention strategies, and clear escalation pathways [22–24, 31]. The present study provides no efficacy or safety evidence for treatment interruption, de-escalation, fixed-duration therapy, or outpatient step-up dosing.

Patient-generated improvement proposals primarily addressed inpatient and outpatient care experience rather than TFIs directly. Quieter rooms, improved food quality, shorter waiting times, predictable scheduling, adequate staffing, empathetic communication, and access to light or low-intensity physical activity may reduce the burden of treatment-related healthcare encounters. Therapy-specific supportive care studies in MM have similarly highlighted the need to assess and manage symptoms such as dysgeusia that can affect nutrition, quality of life, and the overall treatment experience [9, 10]. By reducing this burden, such measures may indirectly influence how restorative patients perceive intervals between active treatment contacts.

This study has several limitations. Its single-center, cross-sectional design limits generalizability, and the tertiary academic setting may not represent community-based populations or health systems with different inpatient and outpatient care patterns. The number approached and reasons for nonparticipation could not be reconstructed from the retained study documentation, precluding calculation of a participation proportion and assessment of nonresponse bias. Data were self-reported and may be affected by recall bias, social desirability bias, or classification error; the inpatient-day item was not validated against administrative records, its wording may not have ensured consistent exclusion of outpatient or day-case encounters, and planned and unplanned admissions were not separated. Current regimen, administration route, treatment line, response status, cytogenetic risk, MRD status, steroid dose and schedule, and validated quality-of-life measures were not captured in sufficient detail for treatment-specific or stratified analysis. Recruitment setting may have influenced responses, but no inpatient-versus-outpatient outcome analysis was conducted. The disease itself was not assessed as a standalone inpatient stressor. The visit-frequency distribution and the non-affirmative categories of the relapse-risk item could not be reconciled to a valid denominator from the archived summary values and were therefore omitted rather than imputed. The exact CIs are wide for several response categories, reflecting limited precision. The study-specific questionnaire did not undergo formal psychometric validation. Preferences may change over the disease course, and stated preferences may differ from decisions made under clinically defined risk-benefit conditions.

Strengths include the mixed-methods design, explicit integration of structured items and qualitative narratives, direct inclusion of patient-generated care-improvement proposals, item-specific denominators, and exact 95% CIs for response proportions. The study provides clinically grounded, descriptive information from a tertiary MM setting while clearly separating preference estimates from claims about clinical safety or efficacy.

## Conclusion

In this single-center mixed-methods survey, participants with MM described intervals between active treatment contacts as potentially important for recovery, autonomy, and restoration of daily routines, particularly in the inpatient treatment context. Regular monitoring was more often reassuring than burdensome, and explicit willingness to accept higher relapse risk for longer intervals was uncommon. Preferred interval length was heterogeneous. Because current regimen, administration route, disease status, and other clinical variables were not captured in sufficient detail, these findings are descriptive and not treatment-specific; they do not establish that longer TFIs are clinically appropriate or safe. Prospective multicenter studies should examine how patient goals, treatment burden, disease risk, care setting, and monitoring preferences can inform preference-sensitive scheduling and monitoring approaches, with prospective evaluation of their safety and feasibility.

## Supplementary information

Below is the link to the electronic supplementary material.

ESM 1 (DOCX 49.9 KB)

## Data Availability

The data supporting the findings of this study are available from the corresponding author upon reasonable request.
