# Supplementary material for: Patient preferences for treatment‑free intervals in multiple myeloma: mixed‑methods insights on monitoring trade‑offs and supportive care improvements
Source: Support Care Cancer. 2026 Oct 2;34(10):1050. doi: 10.1007/s00520-026-11255-z (PMC13633344; doi:10.1007/s00520-026-11255-z)
Supplement: Supplementary file 1 [file 520_2026_11255_MOESM1_ESM.docx]

**Supplementary Table 1: Demographic and clinical characteristics of patients with MM**

| **Characteristic** | **Category** | **Frequency (%)** |
| --- | --- | --- |
| **Age (years)** | Median (IQR) | 67.2 (60.8–72.3) |
| **Gender** | Male | 64 (64%) |
|  | Female | 36 (36%) |
| **Marital Status** | Single | 2 (2%) |
|  | Married/Partnership | 92 (92%) |
|  | Divorced/Partnership Dissolved | 4 (4%) |
|  | Widowed | 2 (2%) |
| **Highest Educational Level** | No school leaving certificate | 0 (0%) |
|  | Basic school leaving certificate | 17 (17%) |
|  | Intermediate school leaving certificate | 31 (31%) |
|  | University entrance qualification | 20 (20%) |
|  | Other | 32 (32%) |
| **Employment Status** | Student | 0 (0%) |
|  | Employed | 19 (19%) |
|  | Self-employed | 5 (5%) |
|  | Homemaker | 3 (3%) |
|  | Unemployed | 0 (0%) |
|  | Retired/early retiree | 58 (58%) |
|  | Partially disabled | 6 (6%) |
|  | Other | 9 (9%) |
| **Year of MM Diagnosis** | Median (IQR) | 2019 (2016–2023) |
| **Hospital Days in Prior Year** | Median (IQR) | 31 (12–57) |
| **Survey setting / recruitment setting** | Inpatient | 45 (45%) |
|  | Outpatient | 55 (55%) |

Survey setting indicates where the questionnaire was completed (inpatient admission or outpatient visit). The number approached and reasons for nonparticipation could not be reconstructed from the retained study documentation. Available clinical variables are shown; current treatment regimen, administration route, treatment line, response status, cytogenetic risk, MRD status, and validated quality-of-life scores were not captured in sufficient detail for stratified reporting.

**Supplementary Table 2: Importance of TFIs for inpatient and outpatient treatment contexts**

| **Importance rating (Inpatient)** | **n/N; % [exact 95% CI]** | **Importance rating (Outpatient)** | **n/N; % [exact 95% CI]** |
| --- | --- | --- | --- |
| 0 (Not important) | 9/100; 9.0% [4.2-16.4] | 0 (Not important) | 16/99; 16.2% [9.5-24.9] |
| 1 | 1/100; 1.0% [0.0-5.4] | 1 | 1/99; 1.0% [0.0-5.5] |
| 2 | 1/100; 1.0% [0.0-5.4] | 2 | 5/99; 5.1% [1.7-11.4] |
| 3 | 3/100; 3.0% [0.6-8.5] | 3 | 5/99; 5.1% [1.7-11.4] |
| 4 | 1/100; 1.0% [0.0-5.4] | 4 | 5/99; 5.1% [1.7-11.4] |
| 5 | 7/100; 7.0% [2.9-13.9] | 5 | 9/99; 9.1% [4.2-16.6] |
| 6 | 6/100; 6.0% [2.2-12.6] | 6 | 7/99; 7.1% [2.9-14.0] |
| 7 | 4/100; 4.0% [1.1-9.9] | 7 | 6/99; 6.1% [2.3-12.7] |
| 8 | 10/100; 10.0% [4.9-17.6] | 8 | 12/99; 12.1% [6.4-20.2] |
| 9 | 9/100; 9.0% [4.2-16.4] | 9 | 3/99; 3.0% [0.6-8.6] |
| 10 (Very important) | 49/100; 49.0% [38.9-59.2] | 10 (Very important) | 30/99; 30.3% [21.5-40.4] |
| **Total N** | **100** | **Total N** | **99** |

Values are n/N; percentage [two-sided exact 95% CI]. Exact CIs were calculated using the Clopper-Pearson method. The inpatient item had N=100 and the outpatient item had N=99.

**Supplementary Table 3: Inpatient stressors, reported as n/N, percentages, and exact 95% CIs**

| **Stressor category** | **n/N; % [exact 95% CI]** | **Illustrative content** |
| --- | --- | --- |
| Separation from family | 27/100; 27.0% [18.6-36.8] | Separation and reduced contact with family were described as burdensome, particularly during longer admissions. |
| Treatment-related symptoms or side effects | 23/100; 23.0% [15.2-32.5] | Reported experiences included bone pain, nausea, taste disturbance, and diarrhea during admission. |
| Fatigue and exhaustion | 17/100; 17.0% [10.2-25.8] | Participants described fatigue related to illness, treatment, travel, or the admission itself. |
| Quality of hospital food | 15/100; 15.0% [8.6-23.5] | Food quality and limited acceptability were described as affecting eating and the inpatient experience. |
| Long waiting times | 14/100; 14.0% [7.9-22.4] | Waiting for rooms, procedures, or other care processes was described as physically and emotionally burdensome. |
| Shared-room experience | 12/100; 12.0% [6.4-20.0] | Noise, limited privacy, and disruption in shared rooms were reported. |
| Communication or empathy gaps | 11/100; 11.0% [5.6-18.8] | Some participants described insufficiently clear communication or interactions perceived as lacking empathy. |
| Climate control | 10/100; 10.0% [4.9-17.6] | Participants mentioned uncomfortable room temperatures or inadequate climate control. |
| Other health-related limitations | 9/100; 9.0% [4.2-16.4] | Additional limitations included immunosuppression-related restrictions or muscle cramps; this category excluded symptoms/side effects and fatigue coded above. |
| Lack of physical activity | 7/100; 7.0% [2.9-13.9] | Restricted movement and associated muscle or joint discomfort were reported. |

Values are n/N; percentage [two-sided exact 95% CI]. Categories are not mutually exclusive. "Other health-related limitations" represents additional limitations not already coded as symptoms/side effects or fatigue. Stress attributable to MM itself was not assessed as a standalone category.

**Supplementary Table 4: Effective coping strategies reported in free-text responses**

| **Coping strategy** | **n/N; % [exact 95% CI]** | **Illustrative content** |
| --- | --- | --- |
| **Support from Family Members** | 56/78; 71.8% [60.5-81.4] | Family members assisted with household responsibilities, bills, chores, or repairs during admission. |
| **Delegation** | 43/78; 55.1% [43.4-66.4] | Tasks were delegated to relatives or paid help. |
| **Prior Preparation** | 33/78; 42.3% [31.2-54.0] | Participants organized or delegated tasks before admission to reduce disruption. |
| **Time Management** | 27/78; 34.6% [24.2-46.2] | Time between treatment-related contacts was used to catch up on household tasks. |
| **Organizational Skills** | 23/78; 29.5% [19.7-40.9] | Planning and organization were described as reducing stress related to absence from home. |
| **Online Management** | 19/78; 24.4% [15.3-35.4] | Online banking, bill payment, and other digital tools supported task management during admission. |

Categories are based on coded free-text responses from patients who provided evaluable information on coping strategies (N=78). Values are n/N; percentage [two-sided exact 95% CI]. Categories are not mutually exclusive because participants could mention more than one coping strategy.

**Supplementary Table 5: Factors affecting maintenance-therapy attendance, based on coded free-text responses**

| **Factor** | **n/N; % [exact 95% CI]** | **Illustrative content** |
| --- | --- | --- |
| **Logistical Barriers** | 27/84; 32.1% [22.4-43.2] | Distance, traffic, and unreliable public transport were described as barriers; some participants preferred care closer to home. |
| **Health-Related Factors** | 23/84; 27.4% [18.2-38.2] | Pain, polyneuropathy, physical decline, and cumulative strain complicated travel and attendance. |
| **Psychological and Social Factors** | 18/84; 21.4% [13.2-31.7] | Emotional burden, isolation, stress, and impersonal interactions were described; empathetic communication could mitigate these experiences. |
| **Professional and Personal Responsibilities** | 10/84; 11.9% [5.9-20.8] | Work, long-term plans, family duties, and caregiving responsibilities complicated regular attendance. |
| **Financial Costs** | 8/84; 9.5% [4.2-17.9] | Transport expenses and time away from work added financial burden. |

Categories are based on coded free-text responses from patients who provided evaluable information on factors affecting maintenance-therapy appointments (N=84). Values are n/N; percentage [two-sided exact 95% CI]. Categories are not mutually exclusive because participants could mention more than one factor.
